# Long-Term Impairment of Sound Processing in the Auditory Midbrain by Daily Short-Term Exposure to Moderate Noise

**DOI:** 10.1155/2017/3026749

**Published:** 2017-05-14

**Authors:** Liang Cheng, Shao-Hui Wang, Kang Peng, Xiao-Mei Liao

**Affiliations:** ^1^School of Life Sciences and Hubei Key Lab of Genetic Regulation and Integrative Biology, Central China Normal University, Wuhan 430079, China; ^2^School of Psychology and Key Laboratory of Adolescent Cyberpsychology and Behavior, Ministry of Education, Central China Normal University, Wuhan 430079, China; ^3^Institute of Public Health and Molecular Medicine Analysis, Central China Normal University, Wuhan 430079, China

## Abstract

Most citizen people are exposed daily to environmental noise at moderate levels with a short duration. The aim of the present study was to determine the effects of daily short-term exposure to moderate noise on sound level processing in the auditory midbrain. Sound processing properties of auditory midbrain neurons were recorded in anesthetized mice exposed to moderate noise (80 dB SPL, 2 h/d for 6 weeks) and were compared with those from age-matched controls. Neurons in exposed mice had a higher minimum threshold and maximum response intensity, a longer first spike latency, and a higher slope and narrower dynamic range for rate level function. However, these observed changes were greater in neurons with the best frequency within the noise exposure frequency range compared with those outside the frequency range. These sound processing properties also remained abnormal after a 12-week period of recovery in a quiet laboratory environment after completion of noise exposure. In conclusion, even daily short-term exposure to moderate noise can cause long-term impairment of sound level processing in a frequency-specific manner in auditory midbrain neurons.

## 1. Introduction

Noise pollution is recognized as a serious human health problem [1–3]. Normal physiological functions, including those in the auditory system, can be impaired or damaged by exposure to environmental noise [4–9]. Numerous studies have shown that high-intensity noise exposure can damage hair cells in the cochlea [10, 11], increase the threshold of hearing sensitivity [12, 13], and induce hearing loss or tinnitus [14, 15]. High-intensity noise also can alter the normal neural coding processes of auditory signals in individual nuclei along the auditory pathway, including the cochlear nucleus, inferior colliculus (IC), and auditory cortex [14, 16–20]. However, most people are exposed to moderate levels of environmental noise during daily life, rather than high-intensity noise. Further, although the effects of high-intensity noise exposure on hearing have been studied extensively, the potential effects of moderate noise remain unclear.

Recent animal studies have shown that moderate noise exposure can impair cortical processing of acoustic inputs, intensity and frequency domains, as observed with intense noise [7, 19, 21–24]. However, these studies have largely focused on daily long-term or persistent exposure to moderate noise. By contrast, for most of the population, the duration of daily exposure to noise is limited to a few hours, for instance, exposure to traffic noise on the way to the office. Whether exposure to moderate noise for a few hours per day (daily short-term exposure) can also impair the auditory functions in central auditory system, or just induce a weak effect that quickly recovers, has not been addressed. Thus, in the present study, we exposed mice to moderate level noise (80 dB SPL) for 2 h/day for 6 weeks and then assessed sound processing of IC neurons immediately after or at 12 weeks after cessation of noise exposure. In addition, the mammalian nervous system, including the auditory system, undergoes rapid and progressive structural and functional maturation during early development and may be more susceptible to environment factors such as noise [5–7, 25]. Thus, juvenile mice were chosen as experimental subjects.

## 2. Materials and Methods

All experiments were conducted with the approval of the Institutional Animal Care and Use Committee of Central China Normal University.

### 2.1. Animals and Noise Exposure

Forty-four 3-week-old healthy mice (*Mus musculus*, KM, 9–12 g, b. wt.) were used in the present study. These mice were purchased from the Center for Disease Control and Prevention of Hubei province of China. All mice were housed in plastic cages on natural light cycles with free access to water and food, and ambient temperature was maintained at 20–25°C. Twenty-two of all mice were exposed randomly to noise at 80 dB SPL for 2 h/day over 6 weeks. The remaining mice were reared in standard condition without exposure to noise. Immediately following the cessation of exposure, eleven exposed mice were tested with the sound processing properties of neurons in the IC. The other eleven exposed mice were tested after 12 weeks of recovery in a quiet laboratory housing condition. As the different testing time between exposed mice resulted in an age disparity, an age-matched control (*n* = 11) for each test group was required.

The white noise was continuously generated by a noise generator (Nanjing University, Jiangsu, China) with a frequency of 10–10,000 Hz. Before being delivered to mice, it was amplified by a power amplifier (custom-made amplifier) to 80 dB SPL. Noise level was measured with a sound level meter (2610, Brüel & Kjær, Nærum, Denmark) with the microphone placed at the mice location.

### 2.2. Animal Surgery

The surgical procedures were basically the same as described in our previous studies [26–28]. Briefly, after being anesthetized with Nembutal (60–90 mg/kg b. wt.), mouse was glued to a flat head nail on its exposed skull with acrylic glue and dental cement. Exposed tissue was treated with an antibiotic (Neosporin) to prevent any pain and inflammation. Then, mouse was secured to an aluminum plate inside a sound-proof room (temperature 28–30°C) with head immobilized by a set of screws. A small hole (diameter: 200–500 *μ*m) was made in the skull above IC for glass pipette electrode insertion (BF-150-75, WPI, USA; 2 M NaCl, tip diameter: <1 *μ*m, impedance: 5–10 MΩ) to record sound-activated responses. The depths of recorded IC neurons were read directly from the scale of a microdrive (David Kopf, model 640, CA, USA). A local anesthetic (lidocaine) was applied during experiment to reduce any possible pain.

### 2.3. Acoustic Stimulation and Recording of Neuron Response

Continuous sine waves were generated from a function generator (GFG-8016G, Good Will Inst Co. Ltd, Bayan Lepas, Penang, Malaysia) before being formed into pure tone pulses (40 ms with 5 ms rise-decay times). Tone pulses were delivered at 2 pulses/s driven by a stimulator (Model SEN-7203, Nihon Kohden Co, Tokyo, Japan). The tone pulses were then amplified (custom-made amplifier) after passing a decade attenuator (LAT-45, Leader, Yokohama, Japan). Finally, the pure tone pulses were fed into a small loudspeaker (AKG model CK 50, 1.5 cm in diameter, 1.2 g, frequency response 1–100 kHz) which was placed 30 cm away from the mouse ear and 60° contralateral to the recording site. The loudspeaker was calibrated by using a measuring amplifier (2610, B&K, Denmark) with a 1/4-inch microphone (4939, B&K, Denmark) at the mouse's ear. The output of the loudspeaker was expressed in decibel sound pressure level (dB SPL) in reference to 20 *μ*Pa root mean square.

**Figure 1:**
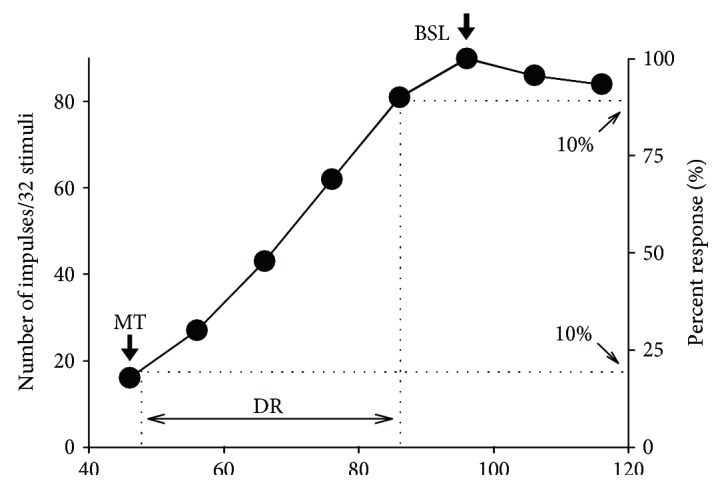
The rate-amplitude function of a representative neuron. The dynamic range (DR) is defined as the amplitude range corresponding to the number of impulse that was 10% below the maximum to 10% above the minimum (indicated by dotted lines). Minimal threshold (MT) and best stimulus level (BSL) are indicated by down arrows. The best frequency (BF, kHz), MT (dB SPL), and recording depth (*μ*m) were 18.6, 48, and 1280.

Upon isolation of an IC neuron with 40 ms pure tone pulses, its threshold at each responsive frequency was determined by changing the sound amplitude which on average elicited 50% response probability from the neuron. The sound frequency that elicited the neuron's response at the lowest amplitude was defined as the best frequency (BF). The threshold at the BF was defined as the minimal threshold (MT). The rate-level functions were then plotted with firing rates obtained at the MT and 10 dB increments above the MT with 40 ms BF sound. The best stimulus level was defined as the specific stimulus level which elicited a maximal firing rate from a neuron. The dynamic range (DR) was defined as the level range from 10% below the maximal to 10% above the minimal firing rates and as middle value of DR, respectively. The slope of a rate-level function was obtained by dividing the percent change in the firing rates within the dynamic range by the dynamic range and expressed in %/dB (Figure 1). The frequency tuning curves were then plotted with firing rates obtained at different frequencies (1–32 kHz, 1 kHz increment) with 40 ms sound at the best stimulus level.

### 2.4. Data Collection and Analysis

Action potentials were amplified and then sent to a computer for acquisition of poststimulus time histograms (PSTH) (bin width: 500 *μ*s; sampling period: 150 ms) to 32 stimuli. The total firing rate in each histogram was used to quantify the neuron's response under each stimulation condition. All data were processed and plotted using the Sigam Plot 2000 (Systat Software, San Jose, CA, USA) and then quantitatively examined and statistically compared using the SPSS 13.0 (one-way ANOVA and Student's *t*-test at *P* < 0.05) (SPSS, Chicago, IL, USA).

## 3. Results

The response parameters of 108 and 121 IC neurons were evaluated either immediately or 12 weeks after the end of noise exposure, respectively. The neural responses were compared with those from 102 and 117 IC neurons in age-matched control animals, respectively. The recording depth of neurons evaluated immediately or at 12 weeks after noise exposure was similar to that of age-matched controls (Table 1). Thus, our findings can be attributed to the effects of noise exposure rather than any sampling bias.

### 3.1. Effect of Noise Exposure on Neuronal Response at Different Frequencies

**Figure 2:**
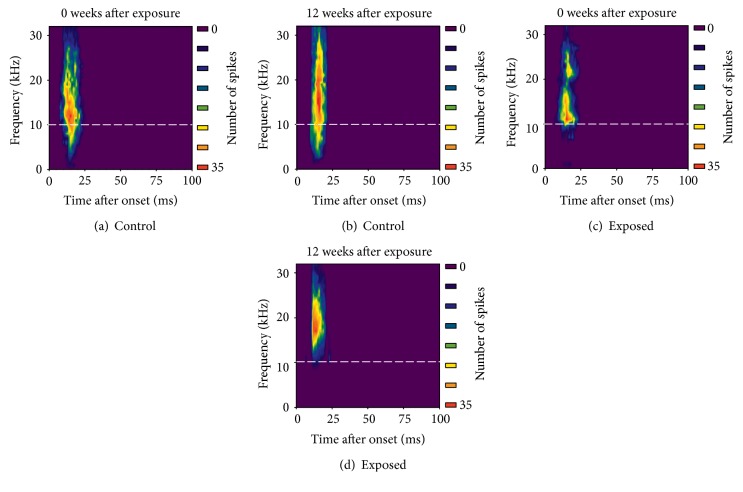
Representative time-frequency tuning of IC neurons from exposed mice and their age-matched unexposed control measured immediately after (a, c) or at 12 weeks after cessation of noise exposure (b, d). Neurons (a) and (c) were from a region of IC tuned to 11 kHz; neurons (b) and (d) were from a region of IC tuned to 18 kHz. Dashed white lines show the frequency of 10 kHz.

**Figure 3:**
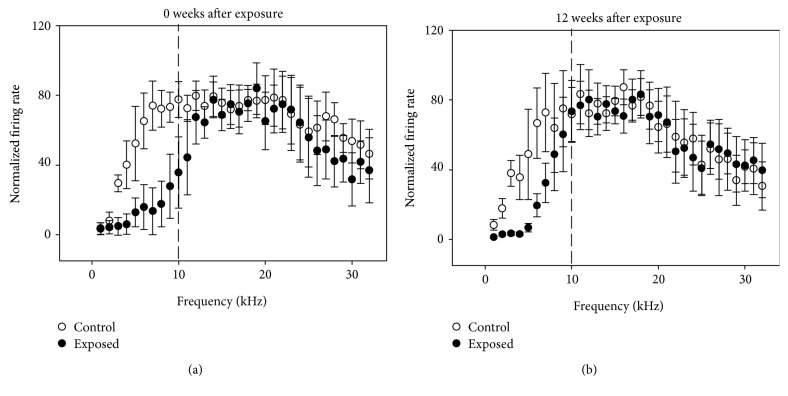
Averaged frequency tuning curves of IC neurons from exposed mice (filled circles) and their age-matched unexposed control (unfilled circles) measured immediately after (a) or at 12 weeks after cessation of noise exposure (b). Each frequency tuning was normalized on their maximum firing rates before averaging at different frequencies (1–32 kHz, 1 kHz increment). The standard deviation is shown by bars on and under each circle. Vertical dashed lines show the frequency of 10 kHz.

Because of the narrow bandwidth frequency spectrum of noise (1–10 kHz) used in this study, neuronal frequency tuning was assessed to identify differences in the effects of noise on the neuronal responses to frequency within and outside of the exposure frequency range. The representative time-frequency tuning of IC neurons from noise-exposed mice and age-matched unexposed control mice is shown in Figure 2. In noise-exposed mice with no recovery, there was a clear decrease in the spike of IC neurons at a frequency below 10 kHz (Figures 2(a) and 2(c)). There was a significant decrease in the averaged frequency-response curves of IC neurons from exposed and unexposed mice below 10 kHz (*P* < 0.01; Figure 3(a)). However, there were no differences above 10 kHz. These data suggest that noise exposure induced a frequency-specific decrease in the response of midbrain auditory neurons over the frequency range of noise exposure. Similar results were found in mice at 12 weeks after the end of noise exposure (Figures 2(b) and 2(d); Figure 3(b)).

### 3.2. Effect of Noise Exposure on Minimal Threshold and Best Stimulus Level

**Figure 4:**
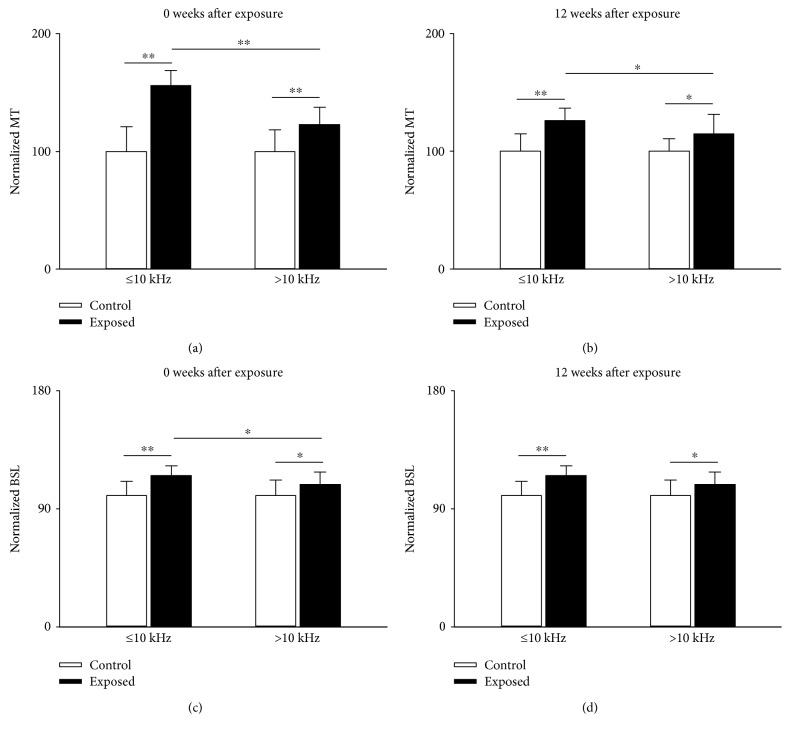
Comparison of minimal threshold (a, b) and best stimulus level (c, d) of IC neurons from exposed mice (filled columns) and their age-matched unexposed control (unfilled columns) measured immediately after (a, c) or at 12 weeks after cessation of noise exposure (b, d). The averaged MT and BSL from exposed mice were normalized on that from unexposed control after averaging. Bars atop of column are the standard deviation. ^∗^*P* < 0.05, ^∗∗^*P* < 0.01, Student's *t*-test.

Because the frequency-specific effects of noise occurred over the exposure frequency range (Figures 2 and 3), the minimal threshold and best stimulus level were separately evaluated in two nonoverlapping bands of BFs (≤10 kHz or >10 kHz) to identify differences between neurons with BFs within or outside of the exposure frequency range. Neurons of noise-exposed mice had a higher minimal threshold (Figure 4(a)) and best stimulus level (Figure 4(c)) compared with control neurons in both BF bands when examined immediately after noise exposure. However, neurons with a BF ≤ 10 kHz showed a greater average increase in the minimal threshold and the best stimulus level than neurons with a BF > 10 kHz. (Figures 4(a) and 4(c)). At 12 weeks after noise exposure, the minimal threshold and best stimulus level in neurons with a BF ≤ 10 kHz and >10 kHz, respectively, remained significantly higher than those of control neurons (Figures 4(b) and 4(d)). Further, the minimal threshold between neurons in these two BF bands (≤10 kHz and >10 kHz) remained significantly different (Figure 4(b)). These data suggest that daily short-term noise exposure may cause long-term increases in the minimal threshold and best stimulus level of IC neurons, which is higher in neurons with a BF within the exposure frequency range.

### 3.3. Effect of Noise Exposure on First Spike Latency

**Figure 5:**
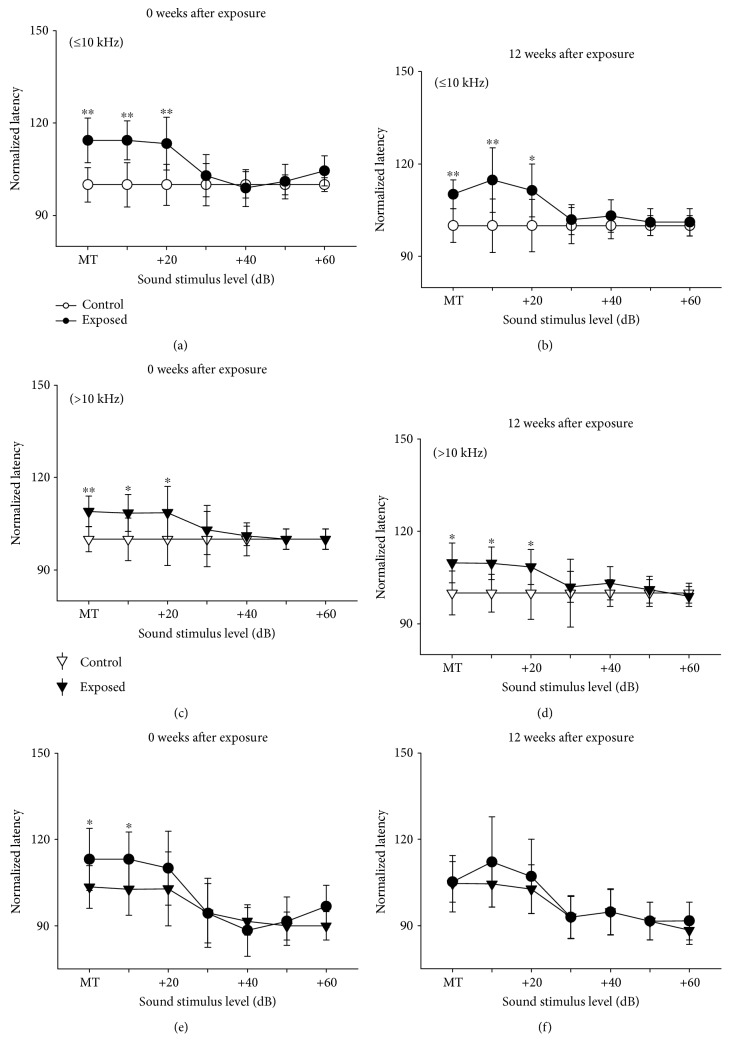
Comparison of latencies of IC neurons from exposed mice (filled circles and triangles) and their age-matched unexposed control (unfilled circles and triangles) measured immediately after (a, c, and e) or at 12 weeks after cessation of noise exposure (b, d, and f). The averaged latency from exposed mice was normalized on that from unexposed control after averaging at different sound stimulus levels. Bars on and under circles show the standard deviation. ^∗^*P* < 0.05, ^∗∗^*P* < 0.01, Student's *t*-test.

The first spike latency of neurons was measured using BF sound at a variable intensity level. At each stimulus level, the averaged latencies in noise-exposed and control mice were compared and the analysis was performed separately in two nonoverlapping BF bands (≤10 kHz or >10 kHz). The normalized averaged latencies of IC neurons in exposed and control mice in the two BF bands, with different recovery times, are shown in Figure 5. Immediately after noise exposure, the latencies of neurons in exposed mice were significantly longer than those in controls at low-sound stimulus levels (Figures 5(a) and 5(c)). However, the lengthening of latencies in neurons with a BF ≤ 10 kHz was greater than that in neurons with a BF > 10 kHz (Figure 5(e)). At 12 weeks after exposure, the latencies remained abnormal (Figures 5(b) and 5(d)), although there were no differences between neurons with a BF ≤ 10 kHz and >10 kHz (Figure 5(f)). These data suggest that daily short-term exposure to moderate noise can cause a long-term abnormality in latency, which is greater in neurons with a BF in the exposure frequency range.

### 3.4. Effect of Noise Exposure on Dynamic Range and Slope

**Figure 6:**
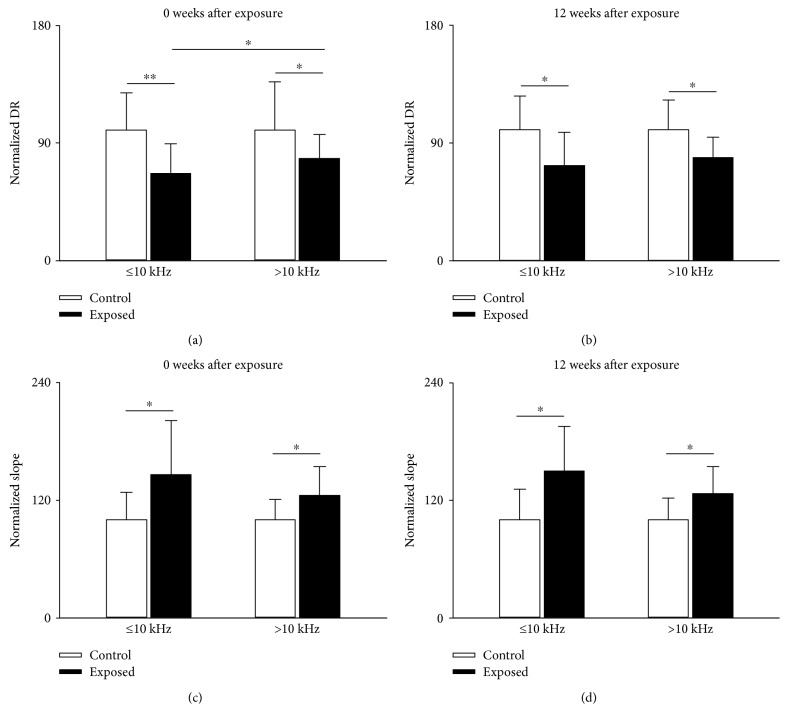
Comparison of DR (a, b) and slope (c, d) of IC neurons from exposed mice (filled columns) and their age-matched unexposed control (unfilled columns) measured immediately after (a, c) or at 12 weeks after cessation of noise exposure (b, d). The averaged DR and slope from exposed mice were normalized on that from unexposed control after averaging. Bars atop of column are the standard deviation. ^∗^*P* < 0.05, ^∗∗^*P* < 0.01, Student's *t*-test.

We also compared the dynamic range and slope of rate-level functions of IC neurons between noise-exposed and unexposed animals at different recovery times (Figure 6). As for the minimal threshold and latency, the analysis of dynamic range and slope of IC neurons was performed separately in two nonoverlapping BF bands (see above). Neurons of exposed mice had a narrower dynamic range and higher slope compared with those of controls at both 0 and 12 weeks after exposure (Figure 6). Note that at 0 week after exposure, neurons in noise-exposed mice with a BF ≤ 10 kHz had a greater decrease in dynamic range than those in neurons with a BF > 10 kHz (Figure 6(a)). These data suggest that daily short-term exposure to moderate noise can cause long-term narrowing in dynamic range and increased slope of neurons, particularly for neurons with a BF in the noise exposure frequency range.

## 4. Discussion

Noise, an unwanted or undesirable sound, has become a part of the human environment [29, 30]. The household electrical appliances at home, the traffic flow on your way to work, and the machines at work are common sources of noise. People living in noisy surroundings have increased risk of diseases that affect the auditory system [10–15, 31]. In the present study, we found that even a short-term exposure to “safe” moderate noise caused a significant and permanent impairment of neuronal response properties in IC neurons in adult animals. Further, this impairment was frequency-specific, with stronger impairment to frequencies within the exposure range.

At birth, the basic topography and connectivity of the auditory system are already present, although neuronal projections are broad and nonspecific [32, 33]. With development, unwanted inhibitory connections are eliminated and the inhibitory axonal arbors and dendritic trees become further restricted and precisely targeted to tonotopically narrower bands [34, 35]. Normally, the overall inhibitory strength in the auditory system decreases as a consequence of decreasing numbers of inhibitory synapses [36]. However, in the present study, noise intervention may have altered this normal developmental process, resulting in a persistently high relative inhibition [37–39]. This may account for the impairment of neuronal responses in IC neurons to noise of frequencies both within and outside of the exposure range. Additionally, a sustained increase in activity of the ≤10 kHz portion of the auditory nerve during noise exposure can cause a homeostatic reduction in excitatory afferent synaptic gain of IC neurons to the ≤10 kHz region after noise exposure [19, 40]. This homeostatic reaction results in further impairment of IC neuron responses to frequencies lower than 10 kHz (i.e., within the exposure frequency range). As a result of the reduction in synaptic gain to the ≤10 kHz region of IC neurons, the inhibitory projections (e.g., lateral inhibition) from this region into the >10 kHz part presumably decreased [41, 42]. This disinhibition may partly balance the neuron-response impairment caused by the abnormal high-level inhibition in the >10 kHz region, which may explain why neurons showed greater impairment in response to frequencies within the noise-exposure range than those outside of the exposure range.

The changes in auditory neuron response properties following noise exposure remain unclear. Bures et al. [20] reported that brief noise exposure during development caused a responsive impairment of IC neurons only in the high-frequency, but not low-frequency, regions of the exposure frequency range. However, Pienkowski and Eggermont [19] reported a decrease in the response of auditory cortex to all frequencies within the noise-exposure range and an increase in response to frequencies outside of the exposure range. In the present study, we found a response impairment of IC neurons to sound frequencies both within and outside of the exposure frequency range. We suggest that these discrepancies may result from differences in the methods used in these studies, including the noise structure, exposure duration, and animal age [19–21]. For example, noise with different parameters performed on animals of different ages may cause different effects on synaptic development and homeostatic rescaling of synaptic gains. Brief exposure to strong noise in young animals may primarily cause changes in synaptic development [20], while long-term noise exposure in mature animals may primarily cause a rescaling of synaptic gains [19]. In the present study, long-term noise exposure was performed on 3-week-old mice (i.e., at the end of the critical developmental period [43, 44]), which likely altered synaptic development at the beginning of the exposure period but caused homeostatic rescaling of synaptic gains later during noise exposure. Nevertheless, overall, these findings suggest that noise exposure can induce impairment of neural responses in the auditory system and that the noise structure, duration of exposure, and subject age are important determinants of the potential effects of noise exposure.

Several studies have examined auditory recovery from temporary impairment caused by persistent/long-term moderate noise exposure. Chang and Merzenich [6] showed that noise-reared rats exhibited characteristic frequency maps and receptive field properties that were recovered to control levels at 10 weeks after returning to standard housing conditions. By contrast, Pienkowski and Eggermont [19] reported persisting changes in tonotopic map organization at 12 weeks after the end of noise exposure. Similarly, we found that the response properties in IC neurons remained abnormal at 12 weeks after the end of noise exposure, indicating that even daily short-term exposure to moderate noise may cause a similar long-term impairment of the auditory system to that induced by persistent exposure [6, 19, 45]. This effect of short-term noise exposure may reflect the stronger negative emotion (e.g., anxiety) induced by random short-term exposure compared with that induced by persistent exposure with emotional adaption. This strong negative emotion may influence auditory function and prolong the recovery from noise-induced changes [46, 47]. Future studies are required to confirm these findings.

It is also possible that the long-term effect of daily short-term noise exposure on the neural responses of IC neurons may develop, at least in part, from the auditory periphery (cochlear), as impairment of temporary threshold shifts or synaptopathy may occur in the cochlear following noise exposure, especially in the developing ear [48, 49]. Future studies are required to determine if the periphery auditory system is also impaired following daily short-term noise exposure by measuring cochlear function.

## 5. Conclusion

In conclusion, the present study demonstrated that even daily short-term exposure to moderate noise may also cause a long-term impairment of sound level processing in a frequency-specific way in auditory midbrain neurons.

## Conflicts of Interest

The authors declare no conflict of interest regarding the publication of this paper.

## Figures and Tables

**Table 1:** Comparison of recording depth of IC neurons between control and exposed mice.

Time after exposure	Depth						*t*-test, *P*
	Control			Noise			
	*n*	Range	Mean ± SD	*n*	Range	Mean ± SD	
0 weeks	108	331~2051	1241 ± 481	102	394~2021	1185 ± 477	>0.05
12 weeks	121	555~2083	1162 ± 396	117	260~2180	1215 ± 503	>0.05
*t*-test, *P*			>0.05			>0.05	
